# Quality of post and core placement by final year undergraduate dental students

**DOI:** 10.1371/journal.pone.0294073

**Published:** 2023-11-09

**Authors:** Khadijah M. Baik

**Affiliations:** Faculty of Dentistry, Oral and Maxillofacial Prosthodontics Department, King Abdulaziz University, Jeddah, Saudi Arabia; King Saud bin Abdulaziz University for Health Sciences, SAUDI ARABIA

## Abstract

The success of endodontic restoration of badly compromised teeth depends on the quality of post and core placement and the extra-coronal restoration. Ensuring that students place posts to acceptable quality standards is therefore essential. The aim of this study was to radiographically evaluate post placement by final year undergraduate dental students and to identify any predictors of performance. Two hundred retrospectively and randomly selected posts placed by sixth year students were evaluated radiographically. Data on student gender; number and quality of radiographs; periapical pathology; tooth location; root canal treatment quality; amount of remaining gutta percha; gap between gutta percha and post; post-to-root width; crown-to-root ratio; and types of core material and crowns were recorded. Four criteria were used to grade post placement quality: (i) amount of remaining gutta percha; (ii) gap between gutta percha and post; (iii) post width to root ratio; and (iv) crown-to-root ratio. Assessments were scored to produce final grades. Data are presented using descriptive statistics and the chi-squared test was used to investigate whether student gender or tooth location were associated with final grade. Post and core quality was acceptable in most cases (97.5% were graded as adequate), with no differences in quality between male and female students nor according to tooth location (anterior, premolar, and molar). Just over half of radiographs were adequate quality (53.5%), while just under half were assessed as less than adequate (46.5%) due to cone cutting, overlap, shortening or elongation, although this did not affect formal assessment of post quality. General outcomes of post and core placement by undergraduate students were good, with few errors that might affect the prognosis and long-term survival of treated teeth. Providing undergraduate dental students with clear guidelines on when and how to take radiographs throughout the procedure may improve the quality of post and core treatment and reduce the risk of multiple unnecessary radiographic exposures. From the clinical perspective, although dental students generally place high quality posts and cores, it remains important to monitor the quality and performance of post placement as this determines the survival of compromised teeth.

## Introduction

The survival of pulpless teeth depends on many factors including the remaining tooth structure, the quality of root canal treatment, the quality of post space preparation and placement, and the quality and timing of extra-coronal restoration. Post space preparation and post cementation are basic skills taught to undergraduates in many dental schools and, although the procedure may seem easy to specialists, it can be challenging for undergraduate dental students. Several aspects of post space preparation have been investigated, including gutta-percha removal techniques, immediate or delayed preparation, the amount of root canal filling remaining, sealer type, and obturation techniques [1–4]. Regardless, post space preparation requires the removal of some of the root canal filling material, which can disturb the apical seal, allowing ingress of bacteria and fluid between the periodontium and root canal that can lead to periodontitis and poorer long-term outcomes [5,6], with an apical seal of 3‒5 mm gutta percha preferred [7–9] and an apical seal <3 mm shown to be unpredictable [10]. The ultimate goal is to provide a well-filled root canal with a three-dimensional seal against bacterial colonization [11], which has a reported success rate of 90% in the absence of periapical lesions [12] but which drops to 40‒65% in cases of inadequate root canal treatment and in the presence of periapical lesions [13,14]. Therefore, post preparation is a critical step in the restoration of badly destroyed teeth that students must master to ensure optimal clinical outcomes for their patients.

To achieve this, students and practitioners must consider several factors during post preparation that have clinically or experimentally been shown to influence outcomes. These include: (i) root canal filling length, where healing is improved when gutta percha is <2 mm from the radiographic apex [15,16]); (ii) post length, the ideal debated but as long as possible without jeopardizing the apical seal of gutta percha [17]; (iii) crown-to-root ratio, a major determinant of the suitability of a tooth to serve as an abutment for a fixed or removable partial denture [18,19], ideally 1:2 but rarely observed clinically, so a 1:1.5 ratio is deemed acceptable for fixed partial denture abutment and a 1:1 ratio is acceptable in a healthy periodontium [18]; (iv) post width to root width ratio, with proportionists advocating post space preparation equal to and exceeding one-third of the root width [20,21], conservationists advocating minimal removal of dentin to ensure easy placement of posts without the presence of undercuts and preservation of the maximum amount of tooth structure [22,23], and preservationists advocating preservation of at least 1 mm of dentin surrounding the circumference of a post to prevent root fracture [24]; and (v) gaps between the post and gutta percha, the size of which influences the rate of periapical disease development, with larger gaps predisposing to disease at one-year follow-up and a gap >2 mm resulting in periapical disease in 70.6% of cases [2].

Four previous studies have examined the quality and type of post and core restorations performed by undergraduate students, and in general the quality of post and core restoration was clinically acceptable [25–28]. However, these studies did not examine predictors of post and core placement quality. Therefore, in addition to investigating the quality of root canal treatment (radiographic post width to root diameter ratios, presence or absence of a gap between posts and the remaining gutta percha, the crown-to-root ratio, and the length of the remaining gutta percha acting), this study also examined whether there were predictors of quality that could guide the focus of undergraduate education in this important area.

## Methods

The Research Ethics Committee of the Faculty of Dentistry at King Abdulaziz University Dental Hospital (KAUDH) approved the study protocol (KAUDH; Ref No. 18-12-19). Consent was waived due to the retrospective nature of the study.

Radiographs of 200 posts placed by sixth year students attending KAUDH in the 2017/2018 and 2018/2019 academic years were assessed following post insertion and following crown cementation. Inclusion criteria were male and female students aged 18 and above in their sixth year of studies performing post and core procedures, and exclusion criteria were cases with incomplete root formation, endodontic retreatments, radiographic obstruction, and those previously treated surgically by apicectomy.

All radiographs were digitally saved on an R4 e-filing system (Kodak Dental Systems, v3.1.8). Of 843 radiographs of patients receiving post and core treatment separated into those performed by male and female students, 200 were randomly selected (100 each from males and females) using the random selection feature in Microsoft Excel. Radiograph quality was assessed as adequate or less than adequate according to the presence or absence of cone cutting, overlapping, elongation, or shortening; however, these features did not affect the formal measurements of quality of root canal treatment.

**Fig 1:**
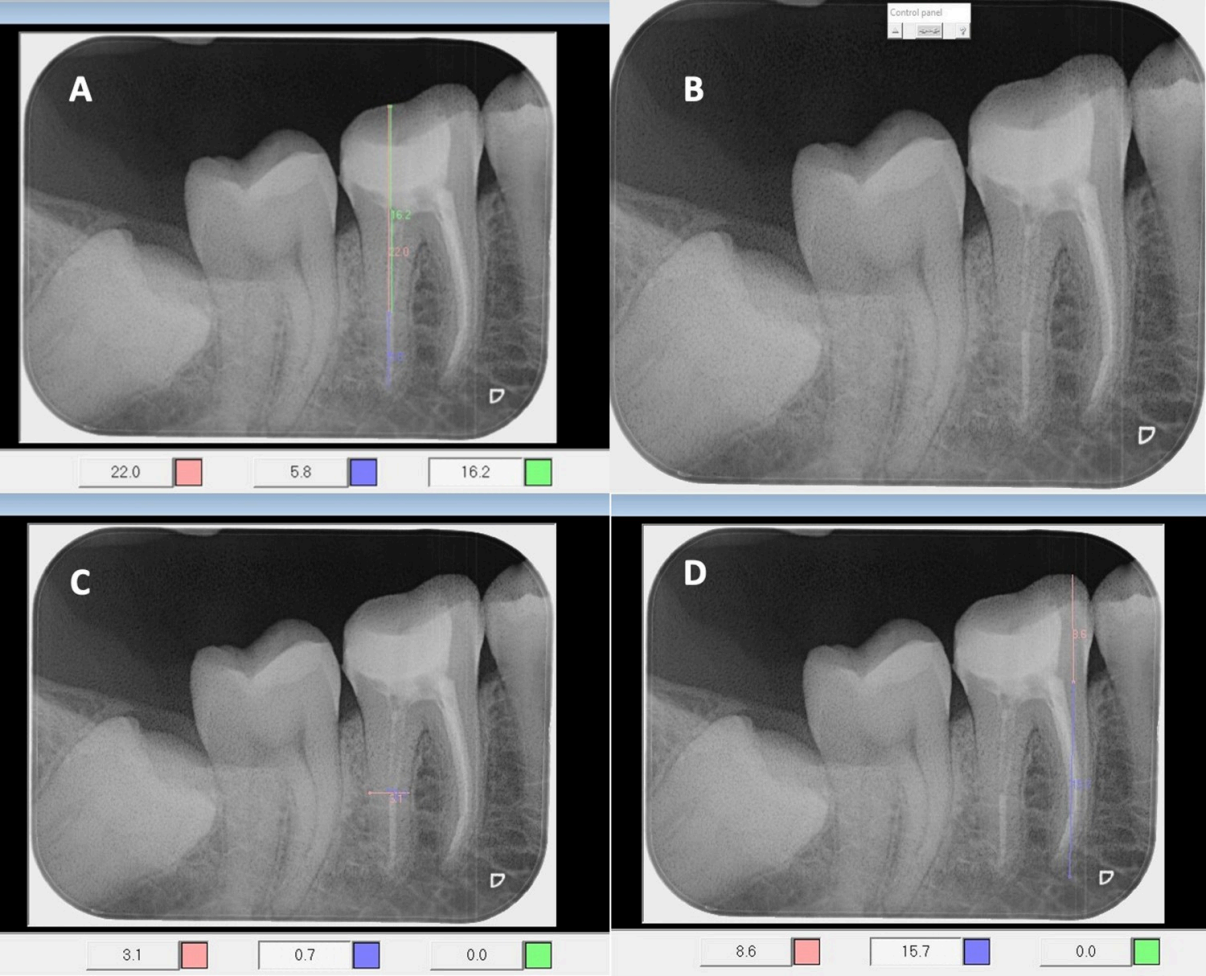
Illustrative measurements made on radiographs. (A) Showing the amount of remaining gutta percha in blue (5.8 mm) and the post space length in green (16.2 mm). (B) Showing no gap between the post and remaining gutta percha. (C) Showing the post width to root width in the middle part. (D) Showing the crown-to-root ratio.

Radiographs were evaluated by two trained dental interns attending KAUDH trained by the principal investigator, with a consultant radiologist supervising radiographic interpretation. The interns were trained to use the calibration feature of the R4. Two calibration sessions were conducted before performing measurements to standardize the procedure for the two dental interns, during which the author explained the correct measurement technique and the two interns practiced until they were reliable and consistent. To check for reliability, inter-rater and intra-rater reliability testing were performed, with measurements retaken 10 days apart for a subset of radiographs. As this process was carried out on 2D digital radiographs on R4 system and using the tools provided by the system, discrepancies were minimal. Illustrative measurements are shown in **Fig 1**.

The following data were collected and recorded in an Excel sheet for analysis: student gender; date of the radiograph; number of radiographs taken; radiograph quality (adequate or less than adequate (cone cut, overlapping, elongated, or shortened)); periapical pathology size; tooth location (anterior, premolar, molar); quality of root canal treatment (adequate: adequate length of root canal filling, homogenous filling with no voids, consistent taper; inadequate: 2 mm short of the radiographic apex or extended beyond the apex, presence of clear voids, inconsistent taper; see [29]); number of posts placed; canal in which the post was placed; type of post used; lengths of the canal and post; amount of remaining gutta percha; gap between the gutta percha and post; post space preparation relative to root width in the apical, middle, and coronal thirds; width of the post relative to the root width in the apical, middle, and coronal thirds; crown-to-root ratio; type of core material used; and type of crown used.

Post success was defined according to the following criteria and as previously [25,30] (see **Table 1**): 1) between 3 and 5 mm gutta percha remaining; 2) absence of a gap between the most apical part of the post and the most coronal part of the gutta percha or presence of a small gap of up to 1 mm; 3) a post width of no more than one-third to one-half the root width; and 4) a crown-to-root ratio of one-half, two-thirds, 1/1. Each quality item was given a score of 0, 1, or 2 (**Table 1**) to generate a total score out of 8, which was then assigned a letter corresponding to the student’s grade according to the percentage score, with F denoting a “fail” (A 90–100%, B 80–89%, C 70–79%, D 60–69%, F <60%).

**Table 1:** Criteria used to define post placement quality (see also [25–27]).

	Poor (0)	Average (1)	Good (2)
**Amount of remaining gutta percha (does not apply to molar)**	1–3 mm	>5.1 mm	3.1–5 mm
**Gap between remaining gutta percha and post**	1.1–2 mm	0.1–1 mm	0 mm
**Post width to root width ratio**	>0.5	0.34–0.5	0.33
	**Reversed (0)**	**Minimal (1)**	**Optimal (2)**
**Crown-to-root ratio**	2/1	1/1	1/2 or 2/3

Post-hoc calculation of the power of the chi-squared test was performed using G*Power software. For α = 0.05, an effect size of 0.3, a sample size of 200, and a maximum df = 3, the power was 0.959.

Data were analyzed using IBM SPSS Statistics v28 software (IBM SPSS Inc., Armonk, NY). Descriptive statistics of frequencies were calculated. The chi-squared test was used to compare student performance according to gender or tooth location (anterior, premolar, molar), and interclass correlations were calculated to assess intra- and inter-observer reliability.

## Results

### Population characteristics

Posts and cores were most commonly placed in premolars (43%) followed by anterior teeth (33.5%) and molars (23.5%). Just over half of students took 1–2 radiographs (57.5%), while 42.5% took three or more radiographs during post preparation and placement (**Table 2**). Just over half of radiographs were adequate quality (52.5%), while just under half were assessed as less than adequate (47.5%) due to cone cutting, overlap, shortening or elongation, although this did not affect subsequent measurements for this study (**S1 Table**).

**Table 2:** Characteristics of the study cohort.

	Characteristic	Frequency	%
Tooth location	Anterior	67	33.5%
	Premolar	86	43%
	Molar	47	23.5%
Number of radiographs taken	1–2 Radiographs	115	57.5%
	≥3 Radiographs	85	42.5%
Quality of radiograph	Adequate	107	52.5%
	Less than adequate	93	47.5%
Quality of root canal treatment	Inadequate	74	37%
	Adequate	126	63%
Type of post used	Fiber Post	173	86.5%
	Custom Made Post	27	13.5%
Canal placement	Single	129	64.5%
	Palatal	30	15%
	Distal	38	19%
	Disto-buccal	1	0.5%
	Buccal	1	0.5%
	Mesial	1	0.5%
Core material used	Composite	172	86%
	Custom-made	28	14%
Crown material used	All ceramic	54	27%
	PFM	131	65.5%
	Composite	13	6.5%
	Metal	2	1%

Root canal treatment was assessed as good quality in 63% of cases and poor quality in 37% of cases. The poor quality of root canal treatment was attributed to the presence of voids (25.5%), a lack of tapering (16.5%), short filling (13%), over obturation (1%), or a combination of multiple factors (**S1 Table**). Students used fiber posts in 86.5% and custom-made posts in 13.5% of cases. Posts were placed in a single canal, in the palatal canal, in the distal canal, in the disto-buccal canal, or in the buccal and mesial canals (**Table 2**). The core material was composite in most cases (86%). Several different crown materials were used (PFM, all ceramic, composite and metal) (**Table 2**). Seventy percent of the periapical pathology ranged between 0 and 3 mm, and 14.5% had no pathology (**S1 Table**).

### Quality of post placement

Inter- and intra-examiner reliability were first examined (**S2 and S3 Tables**). Calculation of the intraclass correlation coefficients (ICCs) showed that both inter- and intra-examiner reliability were excellent, with ICCs >0.95 for all measurements for the former and ICCs >0.95 for all measurements for both examiners for the latter, with the exception of post width to root width ratio for Examiner 1, which was still good at 0.826.

The amount of remaining gutta percha was poor in 1.5%, average in 65.5%, and good in 33% of cases (**Table 3**). The gap between the most apical part of the post and the most coronal part of gutta percha was poor in 5.5%, average in 30%, and good in 64.5% of cases. Post width to root ratio was good in 89.5% and average in 10.5% of cases. The crown-to-root ratio was optimal in 95.5% of cases. Overall, 97.5% of students achieved a pass grade for post space preparation and cementation.

**Table 3:** Quality of post placement.

	Characteristic	Frequency	%
**Amount of remaining gutta percha**	1–3 mm	3	1.5%
	5.1–11.1 mm	131	65.5%
	3.1–5 mm	66	33%
	Total	200	100%
**Gap between remaining gutta percha and post**	1.1–2 mm	11	5.5%
	0.1–1 mm	60	30%
	0 mm	129	64.5%
	Total	200	100%
**Post width to root ratio**	0.34–0.5	21	10.5%
	≤0.33	179	89.5%
	Total	200	100%
**Crown-to-root ratio**	1/1	9	4.5%
	1/2 or 2/3	191	95.5%
	Total	200	100%
**Grade**	A	27	13.5%
	B	94	47%
	C	48	24%
	D	26	13%
	F	5	2.5%
	Total	200	100%

### Associations between grade achieved and other parameters

There were no statistically significant differences in grades between male and female students nor an association between final grade and tooth location (**Table 4**).

**Table 4:** Associations between grade achieved according to student gender and tooth location.

		Grade										Pearson Chi-Square Tests
		A		B		C		D		F		
		Count	%	Count	%	Count	%	Count	%	Count	%	
**Gender Of Doctor**	**Male**	10	10.0%	46	46.0%	29	29.0%	12	12.0%	3	3.0%	.368
	**Female**	17	17.0%	48	48.0%	19	19.0%	14	14.0%	2	2.0%	
**Tooth Location**	**Anterior**	8	11.9%	32	47.8%	15	22.4%	9	13.4%	3	4.5%	.865
	**Premolar**	10	11.6%	40	46.5%	23	26.7%	12	14.0%	1	1.2%	
	**Molar**	9	19.1%	22	46.8%	10	21.3%	5	10.6%	1	2.1%	

## Discussion

Post space preparation and cementation is a standard clinical procedure practiced by general dental practitioners worldwide. Some general practitioners opt to refer such cases to specialist prosthodontists when canal morphology is complex or when they have not performed root canal treatment so are not familiar with canal morphology. However, posts prepared in dental school settings tend to be long-lived (12 years for fiber posts, 10.2 years for prefabricated metal posts, and 11.8 years for case metal post and cores), with survival associated with the percentage of root bone support, cement, and type of final restoration [31]. Additionally, when the remaining dentin thickness is preserved after tooth preparation, post and core restoration survival can be as high as 96% at five years regardless of the type of post and core used [32]. Moreover, the survival of post and core restorations can be influenced by the type of covering prosthesis, location and type of tooth, post and core material, and bone attachment [33].

Here we assessed the quality of post and core placement in sixth year dental students. Overall, outcomes were adequate, consistent with those of previous studies reporting clinically acceptable outcomes from post and core placement in cohorts of dental students [25–28]. Meshni et al. [27] investigated the type and arch of tooth restored, gender of patient, type of post used, and the same quality outcomes as assessed here. In their series, most teeth restored were incisors (41%) rather than premolars (43% in our study), and there were some differences in the quality outcomes: the post to root width was 1/3 in 81% of cases (vs. 89.5% in our series) but the post/crown ratio was 2:1 in (57% and 59%) and 1:1 in (41% and 35%) of male and female cases respectively (vs. 95.5% and 4.5% in our series). The observed remaining amount of gutta percha was 3–5 mm in 55% and more than 5 mm in 29% of cases (vs. 33% and 65.5% in our series). There was no space between gutta percha and post in 75% of cases (vs. 65.5% in our series). Similarly, in another study assessing the quality of 661 posts performed by sixth year undergraduate dental students attending King Abdulaziz University, Saudi Arabia [25], the post placement was again clinically acceptable in most cases, although only 11% of posts met all ideal criteria. A third study from Qassim University, Saudi Arabia, assessed the performance of undergraduate students in placing post and core restorations (the year of study was not specified) [26]. The study examined 421 periapical digital radiographs and assessed type of tooth and arch, type and length of post, and similar quality outcomes. Like our study, most restored teeth were premolars (57.2%), and also similar to our study the post to root ratio was less than a third in the majority of cases (81% vs. 89.5%), only a minority of cases had a crown-to-root ratio of 1:1 (13.8% vs 4.5%), and 61.5% cases had remaining gutta percha >5 mm (cf 65.5% here). Finally, Alshehri et al. reviewed 502 periapical radiographs of posts cemented by undergraduate students and found that preparation and radiographic quality were good in nearly all (≥98%) of cases [28]. Compared with our study, only about a third of cases had a post to root ratio less than 1/3, but 38.8% of cases had remaining gutta percha between 3–5 mm, similar to our result. Therefore, taken together with our data, there is now good evidence that senior dental students are generally proficient at performing post and core restorations, especially when comparing outcomes on similar tooth types.

The metrics assessed in this study are important surrogates of clinical outcomes. Indeed, a well filled root canal provides a three-dimensional seal against bacterial colonization [34], and randomized controlled trials report a primary root canal treatment success rate of 90% if performed under controlled clinical conditions and in the absence of periapical lesions [35]. However, treatment success can decrease to 40‒65% if the root canal procedure is inadequate or in the presence of periapical lesions [36]. The presence of a gap between the most apical part of a post and the most coronal part of the gutta percha is not uncommon and creates a habitat for microorganisms [2]. Gaps may negatively influence microleakage [37], with increased microleakage observed with a gap of 2‒3 mm between the post and the gutta percha compared with either a gap refill with gutta percha or the absence of a gap, at least *in vitro*. Moreover, the gap size may influence the rate of periapical disease development, with larger gaps having a higher rate of disease at one-year follow-up. In addition, a gap >2 mm can cause disease in the periapical area in over two-thirds of cases [2]. An apical seal of 3‒5 mm gutta percha is therefore preferred [8] and an apical seal <3 mm can be regarded as unpredictable [10]. We found that only a third of students achieved an optimal amount of remaining gutta percha of between 3.1 and 5 mm, which was probably due to a two-thirds of treated teeth being anteriorly placed. Placing posts in posterior teeth is determined by the root anatomy, and when the root tapers apically or is severely curved, dentists tend to stop before the curvature to avoid perforation or stripping. Compounding this, 86.5% of posts placed were fiber posts, and since prefabricated posts are supplied at certain lengths, when roots are long the dentist must stop early to leave a coronal segment to retain the core. Hence, leaving >5 mm of gutta percha is justifiable if the post is equal to or more than half the root length, which was frequently the case in our cohort.

Healing is affected by the length of the root canal filling, with an 87‒94% healing rate when the gutta percha extends 0‒2 mm from the radiographic apex [6], 68‒78% when the gutta percha is >2 mm from the apex, and 75‒76% when it extends beyond the radiographic apex [6]. There is also a lower risk of disease with homogenous void-free root canal filling [38].

The appropriate length of post length is still under debate, with some studies suggesting that a longer post is more resistant to fracture and others suggesting no correlation between post length and fracture resistance [39]. Some reports have recommended a post length of three-quarters of the root length or at least half the crown length [40], others have reported a 97% success rate if the post equals the crown length [41], while still others have reported that the minimum post length should be 8 mm [42]. It is known that mechanical stress concentrates at the alveolar crest during mastication, so it has been suggested that the post should always extend beyond the alveolar crest [43]. Longer posts are associated with improved survival rates and the stress distribution may be favorable with longer posts [44], although some studies have reported only minimal differences in stress distribution with different post lengths [45]. *In vitro*, although longer posts required more force to be dislodged, post retention of both short posts (5 mm) and long posts (10 mm) was adequate [46]. Another study reported that varying lengths (3, 5, and 9 mm) of fiber posts cemented in with resin cement did not affect fracture resistance [47]. Post length can therefore be as long as possible without jeopardizing the apical seal of 3‒5 mm gutta percha [17].

The crown-to-root ratio is a major determinant of the suitability of a tooth to serve as an abutment for a fixed or removable partial denture [18]. The crown-to-root ratio describes the radiological relationship between the portion of the tooth within the alveolar bone to the portion of the tooth not within alveolar bone and thus provides information about the amount of alveolar bone support. Although the ideal crown-to-root ratio is 1:2, this is rarely observed clinically, so a 1:1.5 ratio has been suggested as acceptable for fixed partial denture abutment and a 1:1 ratio is acceptable in a healthy periodontium [18]. Nearly all of our students (95.5%) achieved crown-to-root ratios of 1/2 to 1/3. Opinions differ on the ideal post width relative to the root width, with some advocating post space preparation equal to and not exceeding one-third of the root width, while others advocate minimal removal of dentin to ensure easy post placement without undercuts and preserving the maximum amount of tooth structure, with a goal of preserving at least 1 mm of dentin surrounding the post circumference to prevent root fracture [48].

The evaluation of post and core quality was based on radiographs, which are two-dimensional representations of post space preparation and post cementation that do not allow accurate four-dimensional evaluation of the case. The quality of radiographs taken by undergraduate students were often suboptimal, including cone cutting, elongation, shortening, and overlapping, which may have contributed to less-than-optimal interpretation. However, no post was directly involved in a cone cut. Future studies should focus on creating guidelines specifying the exact number of radiographs and when they should be taken during post preparation and cementation. Moreover, a universal grading system of post space preparation/cementation for undergraduate students might help to calibrate grading and highlight critical errors where the success and prognosis of clinical cases is dependent on certain outcomes.

In conclusion, within the limitations of this retrospective study, the quality of post space preparation and post cementation was acceptable (97.5% were graded as adequate), with no differences in quality of post placement according to gender or tooth location (anterior, premolar, and molar). The general outcome of treatment by undergraduate students was good, with few errors that might affect prognosis and long-term survival, probably due to the amount of pre-clinical and clinical teaching in prosthodontics and radiology at the institution. Providing undergraduate dental students with clear guidelines of when to take radiographs throughout the procedure may improve the quality of post and core treatment and reduce the risk of multiple unnecessary radiographic exposures.

## Supporting information

S1 Table Details of the quality of post placement by 6^th^ year dental students. (PDF)

S2 Table Inter-examiner reliability of post assessment criteria. (PDF)

S3 Table Intra-examiner reliability of post assessment criteria. (PDF)

S1 Data Raw data. (XLSX)

S1 File (PDF)
