# Supplementary material for: Quality of post and core placement by final year undergraduate dental students
Source: PLoS One. 2023 Nov 9;18(11):e0294073. doi: 10.1371/journal.pone.0294073 (PMC10635463; doi:10.1371/journal.pone.0294073)
Supplement: S1 Table — (PDF) [file pone.0294073.s001.pdf]

SUPPLEMENTARY TABLE S1

Details of the quality of post placement by 6th year dental students.

	**Characteristic**	b
Quality of radiograph	Cone Cut	17%
	Overlap	31%
	Shortening	3%
	Elongation	1.5%
Quality of root canal treatment	Voids	25.5%
	Not Tapered	16.5%
	Short	13%
	Over Obturation	1%
Post width to root ratio cervical	0.34-0.5	74%
	≤0.33	25.5%
	>0.5	0.5%
	Total	100%
Post width to root ratio apical	0.34-0.5	98.5%
	≤0.33	1.5%
	Total	100%
Post space to root ratio cervical	≤0.33	68%
	0.34-0.5	31.5%
	>0.5	0.5%
	Total	100%
Post space to root ratio middle	≤0.33	87.5%
	0.34-0.5	12.5%
	Total	100%
Post space to root ratio apical	≤0.33	98.5%
	0.34-0.5	1.5%
	Total	100%
Post space cervical	≤1mm	2.5%
	1.1-2mm	88%
	2.1-3mm	9.5%
	Total	100%
Post space middle	≤1mm	21%
	1.1-2mm	78%
	2.1-3mm	1%
	Total	100%
Post space apical	≤1mm	79.5%
	1.1-2mm	20%
	2.1-3mm	0.5%

	Total	100%
Post width cervical	≤1mm	5%
	1.1-2mm	86.5%
	2.1-3mm	8.5%
	Total	100%
Post width middle	≤1mm	22.5%
	1.1-2mm	77%
	2.1-3mm	0.5%
	Total	100%
Post width apical	≤1mm	80%
	1.1-2mm	20%
	Total	100%
Size of periapical pathology	No Pathology	14.5%
	0-3 mm	70.5%
	3.1-5 mm	15%
	Total	100%
