# Supplementary material for: Quality of post and core placement by final year undergraduate dental students
Source: PLoS One. 2023 Nov 9;18(11):e0294073. doi: 10.1371/journal.pone.0294073 (PMC10635463; doi:10.1371/journal.pone.0294073)
Supplement: S2 Table — (PDF) [file pone.0294073.s002.pdf]

SUPPLEMENTARY TABLE S2

Inter-examiner reliability of post assessment criteria

		95% Confidence Interval		
	Intraclass Correlation	Lower Bound	Upper Bound	Value
Amount of remaining gutta percha (does not apply to molar)	0.996	0.985	0.999	590.463
Post width to Root width Ratio	0.953	0.824	0.988	37.777
Gap between remaining gutta percha and post	0.992	0.963	0.998	325.952
Crown to root ratio	1			
