# Supplementary material for: Quality of post and core placement by final year undergraduate dental students
Source: PLoS One. 2023 Nov 9;18(11):e0294073. doi: 10.1371/journal.pone.0294073 (PMC10635463; doi:10.1371/journal.pone.0294073)
Supplement: S3 Table — (PDF) [file pone.0294073.s003.pdf]

SUPPLEMENTARY TABLE S3

Intra-examiner reliability of post assessment criteria

		95% Confidence Interval		
	Intraclass Correlation	Lower Bound	Upper Bound	Value
Amount of remaining gutta percha (does not apply to molar) ***Examiner 1***	0.995	0.982	0.999	438.856
Amount of remaining gutta percha (does not apply to molar) ***Examiner 2***	0.975	0.903	0.994	79.317
Post width to Root width Ratio ***Examiner 1***	0.826	0.446	0.954	10.508
Post width to Root width Ratio ***Examiner 2***	0.962	0.854	0.99	51.088
Gap between remaining gutta percha and post ***Examiner 1***	0.983	0.933	0.996	116.148
Gap between remaining gutta percha and post ***Examiner 2***	0.974	0.9	0.994	76.381
Crown to root ratio ***Examiner 1***	1	1	1	.
Crown to root ratio ***Examiner 2***	1	1	1	.
