# Supplementary material for: Quality of post and core placement by final year undergraduate dental students
Source: PLoS One. 2023 Nov 9;18(11):e0294073. doi: 10.1371/journal.pone.0294073 (PMC10635463; doi:10.1371/journal.pone.0294073)
Supplement: S1 File — (PDF) [file pone.0294073.s005.pdf]

nextgenediting

EDITING CERTIFICATE

This document certifies that one or more of the native
English-speaking editors at Nextgenediting edited the English language, grammar,
punctuation, spelling, and overall style of the document below.

MANUSCRIPT TITLE

Quality of Post and Core Placement by Final Year Undergraduate Dental Students

AUTHORS

Khadijah M. Baik

DATE ISSUED

4th October 2023

CERTIFICATE VERIFICATION KEY

HGRH2-329RG-2KR0N-HH3BR

This certificate may be verified by emailing the Certificate Verification Key to certification@nextgenediting.com. This document certifies that one or more of the doctoral-level native English-speaking editors at Nextgenediting edited the manuscript named above for English language, grammar, punctuation, spelling, and overall style. The research content and the authors' intentions were not altered in any way during the editing process. Documents receiving this certification should be English-ready for publication; however, the author has the ability to accept or reject our suggestions and changes. To verify the final Nextgenediting edited version, please email us at the above address.
If you have any questions or concerns about this edited document, please contact Nextgenediting at enquiries@nextgenediting.com.

Nextgenediting provides a range of editing and manuscript services for researchers and publishers around the world. Our expert doctorate-qualified editors are all native English speakers from the UK's top universities. For more information about our company, services, and partner discounts, please visit www.nextgenediting.com.
